# Femtosecond Circular Photon Drag Effect in the Ag/Pd Nanocomposite

**DOI:** 10.1186/s11671-016-1771-4

**Published:** 2017-01-13

**Authors:** Gennady M. Mikheev, Aleksandr S. Saushin, Viatcheslav V. Vanyukov, Konstantin G. Mikheev, Yuri P. Svirko

**Affiliations:** 1 Institute of Mechanics UB RAS, Izhevsk, Russia 426067; 2 Institute of Photonics, University of Eastern Finland, 80101 Joensuu, Finland; 3 Department of Physics, M.V. Lomonosov Moscow State University, Moscow, Russia 119991

**Keywords:** Photon drag effect, Circular polarization, Circular photocurrent

## Abstract

We report on the observation of the helicity-dependent photoresponse of the 20-μm-thick silver–palladium (Ag/Pd) nanocomposite films. In the experiment, 120 fs pulses of Ti:S laser induced in the film an electric current perpendicular to the incidence plane. The photoinduced current is a linear function of the incident beam power, and its sign depends on the beam polarization and angle of incidence. In particular, the current is zero for the *p*- and *s*-polarized beams, while its sign is opposite for the right- and left-circularly polarized beams. By comparing experimental results with theoretical analysis, we show that the photoresponse of the Ag/Pd nanocomposite originates from the photon drag effect.

## Background

Growing interest to the engineering of the surface or bulk spin-polarized photoinduced currents [1] has attracted attention to the circular photogalvanic (CPGE) [2] and circular photon drag (CPDE) effects [3]. These phenomena, which manifest themselves as conversion of the photon angular momenta to momentum of charge carrier, were extensively studied in two-dimensional (2D) [1, 4–9] and planar [10–14] materials during the last decade.

CPGE can be observed in gyrotropic media lacking inversion centre and mirror symmetry and originates from the imbalanced distribution in the momentum space of the carriers excited when an elliptically polarized beam hits the sample surface [1]. CPGE provided information on the spin–orbit coupling and has been observed in crystalline bismuth silicate (Bi_12_SiO_20_) [15], lead germanate (Pb_5_Ge_3_O_11_) [16], lithium niobate [17], indium nitride (InN) films [10, 11], quantum wells [4, 7, 8, 18–20] and 2D heterostructures [9, 21].

The photon drag effect [22–24] originates from the transferring the momentum from photon to the charge carriers and manifests itself as light-induced *dc* current. In contrast to the photogalvanic effect, it is permitted in both noncentrosymmetric and centrosymmetric media [25]. The CPDE manifests itself as a helicity-dependent current propagating perpendicular to the plane of incidence. It was observed at oblique incidence in quantum wells [3], graphene [6, 26], InSb [12] and bulk tellurium [27]. At the nanosecond excitation, the CPDE has been recently observed in nanoporous gold thin film [13], 2D metallic photonic crystal slabs [28] and Ag/Pd nanocomposite [14, 29, 30]. However, to the best of our knowledge kinetics of the helicity-dependent photoinduced surface currents injected by a single femtosecond laser pulse in metallic nanocomposite has not been studied yet.

In this paper, we report on the excitation of the helicity-dependent photoinduced voltage (PIV) in a 20-μm-thick Ag/Pd nanocomposite film under irradiation with the femtosecond laser pulses at an oblique incidence. We reveal that the relaxation time of the photocurrent generated at the film surface is as long as several nanoseconds, i.e. the photoresponse of the film lasts much longer than the duration of the incident femtosecond pulse. We demonstrate that the polarity and magnitude of PIV can be controlled by the ellipticity of the laser beam as well as by the angle of incidence. By studying the dependence of the PIV on the polarization and the angle of incidence, we demonstrate that it originates from the CPDE.

## Methods

**Fig. 1:**
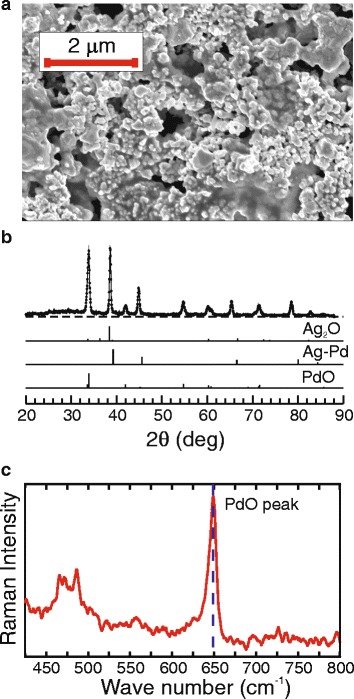
**a** SEM image of a Ag/Pd nanocomposite surface. **b** X-ray diffraction patterns and bar diffraction patterns of detected phases (Cu K_α_). **c** Raman spectrum of the Ag/Pd nanocomposite

Silver–palladium (Ag/Pd) nanocomposites are important for electronics and electronic packaging, such as hybrid microcircuits, multichip modules, packaging for integrated microcircuits and passive electronic components [31]. The optical and electronic properties of the Ag/Pd nanocomposite are varied in a wide range depending on thermodynamics and kinetics of Pd oxidation, Ag diffusion and migration, properties of inorganic and organic additives and other factors. In experiments, we studied Ag/Pd nanocomposites fabricated using a conventional technology described elsewhere [32]. Briefly, a ceramic substrate was coated with a 20-μm-thick layer of paste containing silver oxide (Ag_2_O), palladium and silica nanoparticles and baked at a temperature of *T*_bur_ = 878 K in air [33]. The SEM image in Fig. 1a shows that the obtained Ag/Pd nanocomposite is porous with pore size ranging from 25 to 500 nm. By analysing the X-ray diffraction patterns (see Fig. 1b), we have found that the films consist of the Ag–Pd, PdO and Ag_2_O, with a mass ratio of 80.3:18.7:1.0, respectively. The Ag–Pd solid solution has the fcc lattice with a lattice parameter of 0.4036 nm, while tetragonal PdO crystals belong to space group D_4h_^9^ having the lattice constants *a* = 0.3043 nm and *c* = 0.5337 nm. The lattice parameters of pure Ag and Pd metals are 0.40862 and 0.38902 nm, respectively. Based on the concentration dependence of lattice parameter of the solid solutions, one can estimate that Ag–Pd gives 74% of the total Ag content in the nanocomposite. It has also been found that the size of Ag–Pd and PdO crystallites exceeds 39 and 28 nm, respectively [33]. The X-ray photoelectron spectroscopy measurements revealed that both metallic palladium (binding energy *E*_b_ = 335.4 eV) and palladium oxide (binding energy *E*_b_ = 336.5 eV) are present in the nanocomposite. The Raman spectrum of nanocomposite films obtained using He-Ne laser at 632.8 nm is presented in Fig. 1c. One can see the strong sharp peak with the shift of 649 cm^−1^ in this spectrum. According to [34], this peak is associated with the PdO content in the film. Thus, the Ag/Pd nanocomposite is a cavernous structure composed of metallic Ag–Pd solid solution and semiconductor PdO nanocrystallites.

**Fig. 2:**
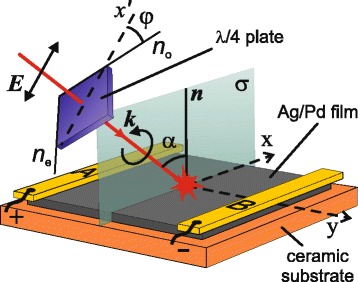
Sketch of the experimental setup containing achromatic quarter-wave plate, Ag/Pd nanocomposite film with electrodes *A* and *B* deposited on the sample’s edges parallel to the plane of incidence σ. The electric field ***E*** in the light beam is parallel to the *x′* axis, ***k*** is the wave vector, ***n*** is the film normal and *α* is the angle of incidence. The plane of incidence *σ* coincides with the *(x*z) plane of the laboratory Cartesian frame; *n*_*o*_ and *n*_*e*_ are fast and slow axis of the quarter-wave plate

The nanocomposite has p-type conductivity 15.2 Ω^−1^ cm^−1^ at the hole density of 9.2 × 10^20^ cm^−3^ and mobility of 1 × 10^−1^ cm^2^ V^−1^ s^−1^. To enable electrical measurements, two parallel silver electrodes *A* and *B* were deposited on the 25 × 25 mm^2^ sample’s edges (see Fig. 2). The inter-electrode resistance and capacitance were 30 Ω and less than 1 pF, respectively.

In our experiments, we employ Ti:S laser operating at a wavelength of *λ* = 795 nm with a pulse repetition rate of 1 kHz. The duration and energy of the laser pulses are 120 fs and 2 mJ, respectively. The sketch of the experimental setup is shown in Fig. 2. The *p*-polarized laser beam with diameter of 4.5 mm passes through an achromatic quarter-wave plate and obliquely incidents onto the film surface. The polarization state of the beam that hits the surface of the Ag/Pd nanocomposite is determined by the angle *φ* between slow axis of the wave plate (*n*_e_) and the polarization azimuth of the incident beam (*x*′). In particular, the beam is left- and right-circularly polarized after quarter-wave plate at *φ* = 45° and −45°, respectively. The plane of incidence (*xz*) is parallel to the electrodes *A* and *B*, which are not irradiated by the laser beam.

The measurements of the PIV was performed by digital oscilloscope with a bandwidth of 200 MHz. The magnitude of the PIV measured at the exposure for 0.2, 1, 2 and 5 s was the same indicating that the heating of the film by the train of the femtosecond pulses with repetition rate of 1 KHz does not influence the photoinduced current. The chosen data acquisition time of as short as 200 ms provides enough data to perform statistical averaging of the signal and allows us to avoid overheating of the nanocomposite. It is worth adding that the PIV measured between electrodes *A* and *B* (see Fig. 2) does not depend on the position of the laser beam on the film surface (providing that electrodes are not irradiated, see also [35]). The large (25 × 25 mm^2^) surface area of the sample allowed us to carry out measurements in incidence angles range ±75° at a laser beam diameter of 4.5 mm.

## Results

**Fig. 3:**
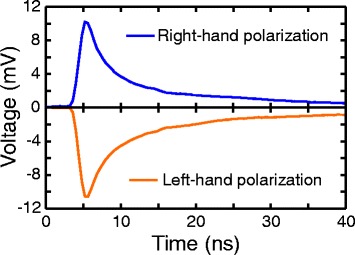
Oscillograms of photo-voltage induced in the Ag/Pd nanocomposite by left-hand (*blue*) and right-hand (*orange*) circularly polarized beams at the incidence angle of *α* = 45°

A femtosecond laser pulse produces an electric current in the film that manifests itself as a unipolar nanosecond PIV pulse between electrodes *A* and *B* that has an opposite polarity for the left- and right-circularly polarized incident beams (see Fig. 3). The rise time *τ*_rise_ of the pulse is determined by the bandwidth of the oscilloscope, while the fall time *τ*_fall_ corresponds to the PIV decay rate. In our experimental conditions, *τ*_rise_ = 1.2 ns and *τ*_fall_ = 22.6 ns are defined with respect to 10 and 90% of the peak value.

**Fig. 4:**
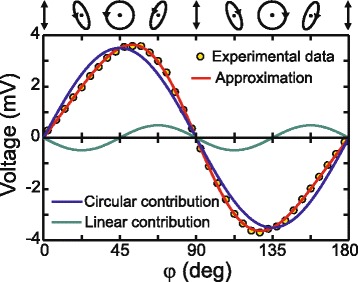
Dependence of the PIV on the quarter-wave plate rotation angle *φ* at *α* = 45° and laser pulse energy of 0.68 mJ. The *red line* shows fitting the experimental data using Eq. (5) at *n =* 1.1 + *i* 1.59 and *μ* = 1.06. The *blue* and *green lines* show the helicity-sensitive and helicity-insensitive contributions, respectively, at *U*_1_ = 3.15 mV and *U*_2_ = 0.49 mV

Figure 4 shows the dependence of the PIV on the quarter-wave plate rotation angle *φ* at the incident angle *α* = 45° and the laser pulse energy of 0.68 mJ. One can observe from Fig. 4 that the signal vanishes when the incident beam is *p*-polarized (i.e. at sin2*φ* = 0). In the independent experiment, we found that PIV is zero also for the *s*-polarized excitation beam. The sign of the PIV is opposite to the sign of the ellipticity of the laser beam, i.e. it is positive at 0 < *φ* < 90° and it is negative at 90° < *φ* < 180°. At *α* = 45°, the peak amplitude of the PIV (see Fig. 4) is well approximated by the following function:

1 $$ {U}_{\mathrm{PIV}}={U}_1 \sin 2\varphi -{U}_2 \sin 4\varphi, $$

where *U*_1_ = 3.51 mV and *U*_2_ = 0.49 mV represent magnitudes of the helicity-sensitive and helicity-insensitive contributions, respectively. It is worth noting that the ellipticity-insensitive part of the photoinduced signal changes the polarity at *φ* = 45°.

**Fig. 5:**
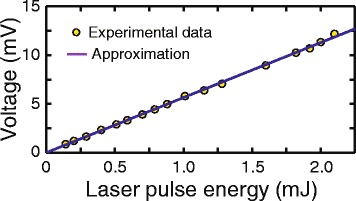
The PIV as a function of the incident pulse power at *φ* = 45° and *α* = 45° (*circles*)

The dependence of PIV on the laser pulse energy is shown in Fig. 5 for the incident angle of *α* = 45° and *φ* = 45° (left-hand polarization). One can observe that PIV is a linear function of *W* with the light conversion efficiency *η* = *U*_PIV_/*W* as high as 5.6 mV/mJ. The analysis of experimental data shows that at nanosecond excitation, the conversion efficiency in Ag/Pd nanocomposite at the excitation wavelength of 795 nm is 2.4 mV/mJ [29], while the conversion efficiency in nanoporous gold films at the excitation wavelength of 600 nm is 0.6 mV/mJ [13]. For comparison, when electrodes are perpendicular to the incidence plane, the conversion efficiency in random nanogold films at excitation wavelength of 530 nm is 0.3 mV/mJ [36].

**Fig. 6:**
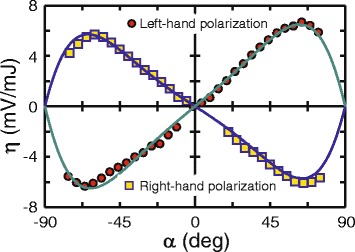
Conversion efficiency *η* as a function of the angle of incidence *α* for the left- (*circles*) and right-hand (*squares*) circularly polarized beams. *Green* and *blue lines* represent results of fitting with Eq. (5) at *n =* 1.1 + *i* 1.59, *μ* = 1.06 and *φ =* ± π/4

Figure 6 shows that the conversion efficiencies *η* ^+^ and *η* ^−^ for the left- and right-circularly polarized excitation beams, respectively, are maximum at *α* ≈ ±60°. It should be noted that the data for 75° < *α* < 90° and −90° < *α* < −75° are not shown because the beam spot size becomes bigger than the lateral film size. One can see from Fig. 6 that *η* ^+^ and *η* ^−^ are odd functions of *α*, *η* ^±^(*α*) = − *η* ^±^(−*α*), and that within the experimental error, the relation *η* ^±^(*α*) = *η* ^∓^(−*α*) also holds.

## Discussion

Under irradiation with nanosecond laser pulse, the temporal profile of the PIV arising due to the photon drag effect reproduces that of the excitation pulse because of the subpicosecond carrier momentum relaxation time (see e.g. [35, 37]). One can observe from Fig. 3 that in Ag/Pd nanocomposite, the 120-fs-long laser pulse generates PIV pulse with a sharp (subpicosecond) front and long (22.6 ns) tail. In the nanosecond experiment [14], the rise time of the PIV signal (3.2 ns) is determined by the excitation pulse, while in the femtosecond experiment, it was restricted by the oscilloscope bandwidth. This indicates that the response time of the nanocomposite lays in the picosecond time scale, i.e. corresponds to the carriers momentum relaxation time. It is worth noting that nanosecond decay time of the PIV signal at the femto- and nanosecond excitations indicates that relaxation time of the photoexcited carriers in Ag/Pd nanocomposite lays in the nanosecond range. Such a long decay time of the photogenerated current may originate from a slow relaxation of charge carriers in the electric field of the Schottky barriers at the interfaces between metallic Ag–Pd and semiconductor PdO crystallites.

Since the film is composed of centrosymmetric Ag–Pd and PdO nanocrystallites, the measured signal cannot be originated from the CPGE [25]. It is worth noting that in the Ag–Pd nanocomposite, the PIV has not shown resonance features in the broad spectral range spanning from 266 to 2100 nm [14, 29]. This indicates that in our experiment, the measured PIV is not originated from plasmon polaritons, which gives rise to the pronounced wavelength dependence of the longitudinal and transversal PIV near plasmon resonance in one- and two-dimensional plasmonic structures [28, 36, 38]. That is in the Ag–Pd nanocomposite, the helicity-dependent photoinduced current directed perpendicular to the incidence plane originates from the CPDE.

The photon drag surface current density on the interface between vacuum and isotropic media can be presented in the following form [39]:

2 $$ {j}_k^{PD}=\frac{\omega }{c}{\xi}_1\mathrm{R}\mathrm{e}{\left[E\times {H}^{\ast}\right]}_k+\mathrm{I}\mathrm{m}\left\{{\xi}_2{E}_l^{\ast}\frac{\partial {E}_k}{\partial {x}_l}+{\xi}_3{E}_k^{\ast}\frac{\partial {E}_l}{\partial {x}_l}\right\}, $$

where subscripts *k* and *l* label Cartesian coordinates (*x*, *y*) on the interface, ***E*** and ***H*** are complex amplitudes of electric and magnetic field in the light wave at frequency *ω* in the medium, *ξ*_1_, *ξ*_2_ and *ξ*_3_ are the complex transport coefficients depending on the excitation wavelength. Equation (2) suggests that the light penetration depth does not exceed the electron mean free path of the medium. In our experimental conditions, the nanocomposite film consists of the Ag–Pd, PdO and Ag_2_O with a mass ratio of 80.3:18.7:1.0, respectively (see Fig. 1b), with 59% of total the Ag content. In silver, the electron mean free pass *l*_σ_ = 57 nm [40], while the light penetration depth *d* = 12 nm [41]. Therefore, we believe that in our experiment, condition *l*_σ_ > *d* is satisfied. It is worth mentioning here that if the electron mean free path is much smaller than the electric field penetration depth, the surface current density should be obtained by integrating the bulk photon drag current over the light penetration depth. In an isotropic medium, the bulk photon drag current lays in the plane of the incidence [42] and it cannot contribute to the PIV measured in our experiment.

Equation (2) allows us to present components of the density of the surface currents propagating along and perpendicular to the plane of incidence in the following form [38]:

3 $$ {j}_x^{PD}\propto \left[{\xi}_1\left(\mathbf{E}{\mathbf{E}}^{\ast}\right)+\mathrm{R}\mathrm{e}\left\{{\xi}_2+{\xi}_3\right\}\left|{E}_y^2\right|\right] \sin \alpha, $$

4 $$ {j}_x^{PD}\propto \mathrm{R}\mathrm{e}\left\{\left({\xi}_2+{\xi}_3^{\ast}\right){E}_x^{\ast }{E}_y\right\} \sin \alpha . $$

One can observe from Eqs. (3) and (4) that photon drag current generated in the plane of incidence (*j*_x_) does not depend on the helicity of the incident beam [13], while that generated in the perpendicular plane (*j*_*y*_) does [13, 14, 29, 30].

In our experimental conditions (see Fig. 2), the PIV is determined by ${j}_y^{\mathrm{PD}}$ and can be presented in the following form:

5 $$ {U}_{\mathrm{PIV}}\propto \mathrm{I}\mathrm{m}\left\{U\left(\alpha \right)\left[1-i \cos 2\varphi \right]\right\} \sin 2\varphi, $$

where

$$ U\left(\alpha \right)=\frac{\left(1+i\mu \right)\sqrt{n^2-{ \sin}^2\alpha } \sin 2\alpha }{\left(\sqrt{n^2-{ \sin}^2\alpha }+{n}^2 \cos \alpha \right)\left(\sqrt{n^{\ast 2}-{ \sin}^2\alpha }+ \cos \alpha \right)},\kern0.24em \mu =\frac{\mathrm{Im}\left({\xi}_2-{\xi}_3\right)}{\mathrm{Re}\left({\xi}_2+{\xi}_3\right)}, $$

*n* is the complex refractive index of the film. Equation (5) implies that the sign of the PIV is opposite for left-hand (*φ* = +45°) and right-hand (*φ* = −45°) circularly polarized excitation beams. It is also worth noting that the helicity-sensitive signal vanishes in purely dielectric medium with real refractive index. In Figs. 4 and 6, solid lines show fitting of the experimental data with Eq. (5) at *n =* 1.1 + *i* 1.59 and *μ* = 1.06.

The incident angle and helicity dependencies of the PIV induced by the femtosecond laser pulses shown in Figs. 4 and 6 resemble those measured at nanosecond excitation [14, 30]. We believe that the Eq. (5) can be also employed to elucidate results obtained in [14]. It is worth noting that the high porosity of the Ag/Pd nanocomposite (see Fig. 1a) prevents direct ellipsometric measurement of the complex refractive index. However, one can observe from Figs. 4 and 6 that Eq. (5) fits well experimental data at complex refractive index *n =* 1.1 + *i* 1.59, which corresponds to the light penetration length of 40 nm. Although this value is higher than that for pure silver (13 nm), the light penetration depth in the composite is smaller than the electron mean free pass in silver (57 nm). This indicates that the condition *d < l*_*σ*_ holds, i.e. the analysis based of Eq. (5) is correct.

It is necessary to mention that in [12] and [13], the origin of the observed helicity-dependent photoinduced currents in centrosymmetric strain-free InSb crystal and a porous gold film, respectively, has not been explained. In contrast, our theoretical and experimental results suggests that CPDE does explain the light-induced surface current in highly conductive porous films.

One can observe from Figs. 4, 5 and 6 that irradiation of the nanocomposite with femtosecond pulses results in the photoresponse with amplitude as high as several mV. By comparing this experimental finding with results obtained for the nanographite [43] and single-walled carbon nanotubes films [35] one may conclude that the magnitude of PIV generated in the Ag/Pd nanocomposite can be increased by suppressing short-circuit currents. This can be done by decreasing the area and thickness of the film and/or reducing the distance between the electrodes. Furthermore, PIV can be obviously increased by amplifying the signal [13], thus allowing one to observe photoresponse at much lower pulse energy. The conversion efficiency can be increased even further by accumulating and averaging the signal opening a way towards application of Ag/Pd nanocomposite in the beam helicity sensors.

## Conclusions

We demonstrate for the first time that the helicity-sensitive transverse photocurrents in Ag/Pd nanocomposite can be generated by a femtosecond light pulse. By comparing results of nanosecond and femtosecond experiments we show that the shorter the laser pulse, the faster the rise time of the helicity-dependent PIV signal, while the fall time of the PIV pulses remains the same. This experimental finding, which evidences a long relaxation time of the photoexcited carriers in the Ag/Pd nanocomposite, allows us to revisit and clarify the results of the nanosecond experiment. The similarity in the angular and polarization dependence of the photoinduced currents clearly show that results of both nanosecond and femtosecond experiments can be explained by the photon drag effect in the metal-semiconductor nanocomposite. We developed a phenomenological theory, which describes results of measurements at the nano- and femtosecond excitation. This theory can also be employed for interpretation of the experiments on the helicity-dependent PIV in centrosymmetric conductive films. The opportunity to tune optoelectronic properties and pronounced polarization dependence of the PIV make Ag/Pd nanocomposite an interesting material for fabrication of helicity-sensitive photon drag photodetectors.

## Abbreviations

Ag/Pd: Silver–palladium
CPDE: Circular photon drag effect
CPGE: Circular photogalvanic effect
PIV: Photoinduced voltage

## References

[1] Ivchenko EL (2002). Circular photogalvanic effect in nanostructures. Physics-Uspekhi.

[2] Asnin VM, Bakun AA, Danishevskii AM, Ivchenko EL, Pikus GE, Rogachev AA (1979). Circular photogalvanic effect in optically active crystals. Solid State Commun.

[3] Shalygin VA, Diehl H, Hoffmann C, Danilov SN, Herrle T, Tarasenko SA, Schuh D, Gerl C, Wegscheider W, Prettl W, Ganichev SD (2006). Spin photocurrents and circular photon drag effect in (110)-grown quantum well structures. JETP Lett.

[4] Ganichev SD, Ivchenko EL, Prettl W (2002). Photogalvanic effects in quantum wells. Phys E.

[5] Yang CL, He HT, Ding L, Cui LJ, Zeng YP, Wang JN, Ge WK (2006). Spectral dependence of spin photocurrent and current-induced spin polarization in an InGaAs/InAlAs two-dimensional electron gas. Phys Rev Lett.

[6] Jiang C, Shalygin VA, Panevin VY, Danilov SN, Glazov MM, Yakimova R, Lara-Avila S, Kubatkin S, Ganichev SD (2011) Helicity-dependent photocurrents in graphene layers excited by midinfrared radiation of a CO_2_ laser. Phys Rev B 84:125429

[7] Yu JL, Chen YH, Jiang CY, Liu Y, Ma H (2011). Room-temperature spin photocurrent spectra at interband excitation and comparison with reflectance-difference spectroscopy in InGaAs/AlGaAs quantum wells. J Appl Phys.

[8] Yu J, Chen Y, Cheng S, Lai Y (2013) Spectra of circular and linear photogalvanic effect at inter-band excitation in In_0.15_Ga_0.85_As/Al_0.3_Ga_0.7_As multiple quantum wells. Phys E 49:92–96

[9] Duan JX, Tang N, Ye JD, Mei FH, Teo KL, Chen YH, Ge WK, Shen B (2013) Anomalous circular photogalvanic effect of the spin-polarized two-dimensional electron gas in Mg_0.2_Zn_0.8_O/ZnO heterostructures at room temperature. Appl Phys Lett 102:192405

[10] Zhang Z, Zhang R, Liu B, Xie ZL, Xiu XQ, Han P, Lu H, Zheng YD, Chen YH, Tang CG, Wang ZG (2008). Circular photogalvanic effect at inter-band excitation in InN. Solid State Commun.

[11] Zhang Z, Zhang R, Xie ZL, Liu B, Li M, Fu DY, Fang HN, Xiu XQ, Lu H, Zheng YD, Chen YH, Tang CG (2009). Wang ZG Observation of the surface circular photogalvanic effect in InN films. Solid State Commun.

[12] Frazier M, Cates JG, Waugh JA, Heremans JJ, Santos MB, Liu X, Khodaparast GA (2009). Photoinduced spin-polarized current in InSb-based structures. J Appl Phys.

[13] Akbari M, Onoda M, Ishihara T (2015). Photo-induced voltage in nano-porous gold thin film. Opt Express.

[14] Mikheev GM, Saushin AS, Vanyukov VV (2015). Helicity-dependent photocurrent in the resistive Ag/Pd films excited by IR laser radiation. Quantum Electron.

[15] Petrov MP, Grachev AI (1979) Photogalvnic effects in bismuth silicate (Bi_12_SiO_20_). Pis’ma v ZhETF 30:18–21

[16] Lemanov VV, Esayan SK, Maksimov AY, Gabrielyan VT (1981) Circular photovoltaic effect in the ferroelectric Pb_5_Ge_3_O_11_. Pis’ma Zh EkspTeor Fiz 34:444–446

[17] Kazanskii PG, Prokhorov AM, Chernykh VA (1985). Direct observation of a circular photocurrent in lithium niobate. Pis’ma Zh Eksp Teor Fiz.

[18] Ganichev SD, Ivchenko EL, Danilov SN, Eroms J, Wegscheider W, Weiss D, Prettl W (2001). Conversion of spin into directed electric current in quantum wells. Phys Rev Lett.

[19] Ganichev SD, Prettl W (2003). Spin photocurrents in quantum wells. J Phys Condens Matter.

[20] Danilov SN, Wittmann B, Olbrich P, Eder W, Prettl W, Golub LE, Beregulin EV, Kvon ZD, Mikhailov NN, Dvoretsky SA, Shalygin VA, Vinh NQ, Van Der Meer AFG, Murdin B, Ganichev SD (2009). Fast detector of the ellipticity of infrared and terahertz radiation based on HgTe quantum well structures. J Appl Phys.

[21] Ma H, Jiang C, Liu Y, Yu J, Chen Y (2013) Helicity dependent photocurrent enabled by unpolarized radiation in a GaAs/Al_0.3_Ga_0.7_As two-dimensional electron system. Appl Phys Lett 102:212103

[22] Gibson AF, Kimmitt MF, Walker AC (1970). Photon drag in germanium. Appl Phys Lett.

[23] Danishevskii AM, Kastal’skii AA, Ryvkin SM, Yaroshetskii ID (1970). Dragging of free carriers by photons in direct interband transitions. Sov Phys JETP.

[24] Wieck AD, Sigg H, Ploog K (1990). Observation of resonant photon drag in a two-dimensional electron gas. Phys Rev Lett.

[25] Glazov MM, Ganichev SD (2014). High frequency electric field induced nonlinear effects in graphene. Phys Rep.

[26] Karch J, Olbrich P, Schmalzbauer M, Zoth C, Brinsteiner C, Fehrenbacher M, Wurstbauer U, Glazov MM, Tarasenko SA, Ivchenko EL, Weiss D, Eroms J, Yakimova R, Lara-Avila S, Kubatkin S, Ganichev SD (2010). Dynamic Hall effect driven by circularly polarized light in a graphene layer. Phys Rev Lett.

[27] Shalygin VA, Moldavskaya MD, Danilov SN, Farbshtein II, Golub LE (2016). Circular photon drag effect in bulk tellurium. Phys Rev B.

[28] Hatano T, Ishihara T, Tikhodeev S, Gippius N (2009). Transverse photovoltage induced by circularly polarized light. Phys Rev Lett.

[29] Mikheev GM, Saushin AS, Zonov RG, Styapshin VM (2014). Spectral dependence of circular photocurrent in silver-palladium resistive films. Tech Phys Lett.

[30] Mikheev KG, Saushin AS, Zonov RG, Nasibulin AG, Mikheev GM (2016). Photon-drag in single-walled carbon nanotube and silver-palladium films: the effect of polarization. J Nanophotonics.

[31] Wang SF, Dougherty JP, Huebner W, Pepin JG (1994). Silver-palladium thick-film conductors. J Am Ceram Soc.

[32] Larry JR, Rosenberg RM, Uhler RO (1980). Thick-film technology: an introduction to the materials. IEEE Trans. Components, Hybrids, Manuf Technol.

[33] Mikheev GM, Saushin AS, Goncharov OY, Dorofeev GA, Gil’mutdinov FZ, Zonov RG (2014). Effect of the burning temperature on the phase composition, photovoltaic response, and electrical properties of Ag/Pd resistive films. Phys Solid State.

[34] McBride JR, Hass KC, Weber WH (1991). Resonance-Raman and lattice-dynamics studies of single-crystal PdO. Phys Rev B.

[35] Mikheev GM, Nasibulin AG, Zonov RG, Kaskela A, Kauppinen EI (2012). Photon-drag effect in single-walled carbon nanotube films. Nano Lett.

[36] Noginova N, Rono V, Bezares FJ, Caldwell JD (2013). Plasmon drag effect in metal nanostructures. New J Phys.

[37] Obraztsov PA, Mikheev GM, Garnov SV, Obraztsov AN, Svirko YP (2011). Polarization-sensitive photoresponse of nanographite. Appl Phys Lett.

[38] Kurosawa H, Ishihara T (2012). Surface plasmon drag effect in a dielectrically modulated metallic thin film. Opt Express.

[39] Gurevich VL, Laiho R (1993). Photomagnetism of metals: microscopic theory of the photoinduced surface current. Phys Rev B.

[40] Crowell CR, Spitzer WG, Howarth LE, Labate EE (1962). Attenuation length measurements of hot electrons in metal films. Phys Rev.

[41] Winsemius P, van Kampen FF, Lengkeek HP, van Went CG (1976). Temperature dependence of the optical properties of Au, Ag and Cu. J Phys F Met Phys.

[42] Normantas E, Pikus GE (1988). E. M. F. induced by entrainment current in a magnetic field. Zh Eksp Teor Fiz.

[43] Mikheev GM, Zonov RG, Obraztsov AN, Volkov AP, Svirko YP (2006). Quick-response film photodetector of high-power laser radiation based on the optical rectification effect. Tech Phys.

